# Amelanotic melanoma with neural lesion simulating leprosy^⋆^

**DOI:** 10.1016/j.abd.2022.12.012

**Published:** 2024-04-23

**Authors:** Andrezza Telles Westin, Sebastião Antônio de Barros Junior, Cacilda da Silva Souza

**Affiliations:** a Division of Dermatology, Department of Internal Medicine, Faculty of Medicine, Universidade de São Paulo, Ribeirão Preto, SP, Brazil; b Department of Pathology and Forensic Medicine, Faculty of Medicine, Universidade de São Paulo, Ribeirão Preto, SP, Brazil

Dear Editor,

Amelanotic/hypomelanotic melanoma (AHM) is a subtype of cutaneous melanoma with little or no pigment on macroscopic inspection and dermoscopic evaluation, or absence of melanin on histopathology. It is a rare entity, with variable frequency, between 0.4% and 27.9%, but possibly underestimated.^1^

The absence of pigmentation and clinical criteria for suspected melanoma, and the morphological variability of AHM possibly lead to erroneous and late diagnosis.^1^, ^2^ AHM can mimic a variety of benign and malignant diseases of different etiologies, whether inflammatory or infectious, in addition to neoplasias.^1^, ^2^

An 81-year-old woman was referred with suspected leprosy due to loss of strength and dropping of her left hand two months before and asymptomatic lesions on her left elbow three years before. The examination showed a group of pink papules and nodules, slightly shiny, not adhered to deeper planes, with polymorphic vessels on dermoscopy, on the side of the elbow (Fig. 1A); a deficit in arm abduction and extension of the left elbow, wrist and fingers; and a palpable 5-cm mass in the left armpit. Magnetic resonance imaging showed an expansive lesion involving the left axillary vascular-nervous bundle (Fig. 1B); electroneuromyography showed severe damage to the radial nerve. Histopathological analysis showed lentiginous proliferation of atypical melanocytes, arranged in confluent nests in the epithelium and in pagetoid migration to the upper layers of the epidermis. The dermis was infiltrated by a neoplasm of pleomorphic cells with vesicular nuclei and basophilic cytoplasm with evident nucleoli, arranged in nests and blocks, compatible with nodular melanoma of the skin (Fig. 2), presenting satellite, and in-transit metastases in the left arm. Regional metastasis was confirmed by biopsy of the mass involving the left axillary neurovascular bundle, thus defining the initial staging in TxN3cM1. Immunohistochemistry was positive for S100, CEA, and Melan-A (Fig. 3), and negative for HMB-45 and tyrosinase. Treatment with interferon was started (3,000,000 IU, three times a week), axillary radiotherapy was refused by family members, and survival was ten months.

**Figure 1:**
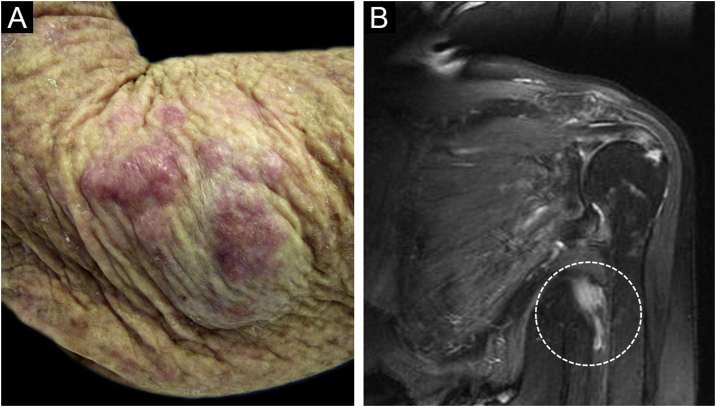
(A) Shiny pink nodules and papules in the left elbow area, representing and in-transit metastases; (B) Magnetic resonance imaging showing a nodular image next to the left axillary vascular-nervous bundle, suggestive of neoplasm.

**Figure 2:**
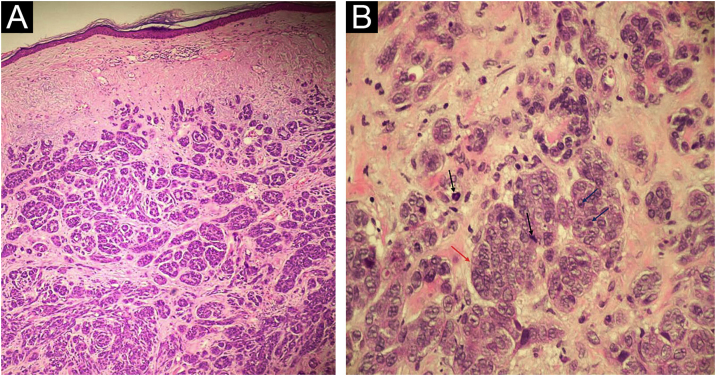
(A) Neoplasm consisting of basophilic cells, forming well-developed nests and masses that infiltrate the dermis, without affecting the epidermis (Hematoxylin & eosin, ×40 and ×100); (B) Cells with eosinophilic cytoplasm and pleomorphic nuclei, vesicular chromatin (red arrow), evident nucleoli (blue arrows) and frequent atypical mitoses (black arrows); (Hematoxylin & eosin, ×40 and ×100).

**Figure 3:**
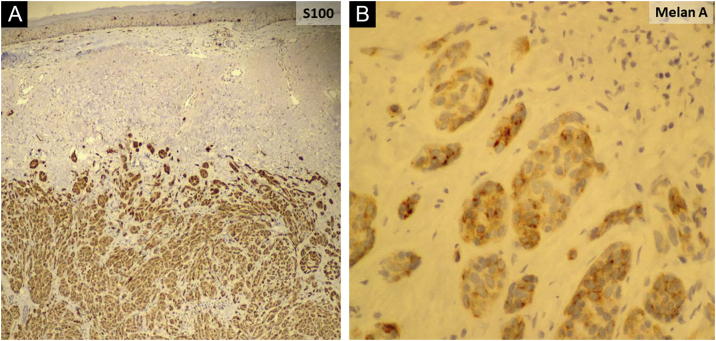
Immunohistochemistry revealing positive staining for S100 (A) and Melan-A (B).

Clinically, red, pink or erythematous lesions represent almost 70% of AHMs, but they may be normochromic.^1^ The papulonodular form predominated in 58% of cases.^1^ Among other clinical forms of AHM, there is the erythematous macule with epidermal changes on skin exposed to the sun, and the normochromic dermal plaque without epidermal changes.^3^ All histopathological subtypes can be found among AHMs: nodular, acral lentiginous and subungual, superficial spreading and lentigo maligna.^1^, ^2^, ^3^, ^4^

Despite the absence of melanin-containing structures, in some cases, it is possible to observe residual pigmentation and a vascular pattern on dermoscopy, not perceptible to the naked eye.^1^, ^5^ Different vascular morphologies have been recognized in AHMs: irregular linear, serpentine (polymorphic), dotted and staple-shaped vessels, as well as milky-red areas, and white structures and lines.^5^ However, the vascular pattern in association with the history and clinical findings, and particularly the histopathological and immunohistochemical analyses are necessary for diagnosis.^1^

Female gender, nodular and unclassified histopathological subtypes, increased Breslow thickness, presence of mitoses, severe solar elastosis, and absence of coexisting nevus have been associated with AHM.^2^ The presence of mitoses, regardless of Breslow thickness, suggests that AHMs may grow more quickly presenting more advanced tumor stages and shorter survival, when compared to pigmented melanoma.^2^ Notably, patients with AHMs were more likely to be misdiagnosed compared to those with pigmented melanomas.^4^ These aspects require a high index of suspicion to potentially minimize late diagnosis at advanced stages of the disease.

## Financial support

None declared.

## Authors’ contributions

Andrezza Telles Westin: Design and planning of the study; data collection, or analysis and interpretation of data; drafting and editing of the manuscript or critical review of intellectual content; collection, analysis and interpretation of data; intellectual participation in the propaedeutic and/or therapeutic conduct of the studied case; approval of the final version of the manuscript.

Sebastião Antônio de Barros Junior: Data collection, or analysis and interpretation of data; collection, analysis and interpretation of data; approval of the final version of the manuscript.

Cacilda da Silva Souza: Design and planning of the study; data collection, or analysis and interpretation of data; drafting and editing of the manuscript or critical review of intellectual content; collection, analysis and interpretation of data; intellectual participation in the propaedeutic and/or therapeutic conduct of the studied case; critical review of the literature; approval of the final version of the manuscript.

## Conflicts of interest

None declared.
